# LEISURE ACTIVITY ENGAGEMENT, SOCIAL CAPITAL, AND URBAN–RURAL INFLUENCES ON IQ: IMPLICATIONS FOR COGNITIVE AGING

**DOI:** 10.1093/geroni/igae098.1812

**Published:** 2024-12-31

**Authors:** Chandra Reynolds, Paige Trubenstein, Shandell Pahlen, Robin Corley, Sergio Rey, Sally Wadsworth

**Affiliations:** University of Colorado Boulder, Boulder, Colorado, United States; Angelo State University, San Angelo, Texas, United States; University of Colorado Boulder, Boulder, Colorado, United States; University of Colorado Boulder, Boulder, Colorado, United States; San Diego State University, San Diego, California, United States; University of Colorado Boulder, Boulder, Colorado, United States

## Abstract

Cognitive health interventions often highlight individual or environmental factors, with research perspectives often siloed between psychological and demography disciplines. With key interest in understanding the rural-urban health divide, we examined how behavioral engagement and geospatial features influence cognitive functioning in individuals approaching midlife. The Colorado Adoption/Twin Study of Lifespan behavioral development and cognitive aging (CATSLife) (N=1201, Mage=33.17 years, range=28.05 to 49.33) offers a unique opportunity to evaluate whether cognitive performance, as indexed by IQ, is associated with engagement in cognitively demanding activities and county-level indices of social capital (SCI; i.e., the availability of social networks and resources) and rurality. To understand the rural-urban health divide, we tested whether activity-IQ or SCI-IQ associations were moderated by rurality. We replicated the known influence of cognitive engagement on IQ, irrespective of locale. A major finding, however, was the cognitive differences observed between rural and urban residents after considering activity engagement. We found rural individuals’ IQ performance was less sensitive to the fluctuations of social capital. Moreover, higher IQ scores among urban residents were only revealed when SCI was high; when SCI was low, urban residents performed worse relative to their rural counterparts. While cognitive engagement had a main effect on IQ, the interaction between SCI and rurality revealed contextually meaningful differences in cognitive functioning. Factors contributing to cognitive health are critical to understand, and our study reveals the interplay of lifestyle and environmental factors on cognitive functioning decades before onset of neurocognitive dysfunction, with implications for cognitive maintenance at midlife and beyond.

